# A skin isolate of *Micrococcus luteus* negates the *Staphylococcus aureus-*induced release of type 2 cytokines from keratinocytes

**DOI:** 10.3389/fimmu.2026.1711723

**Published:** 2026-01-28

**Authors:** Abigail E. Elias, Joanne L. Pennock, Andrew J. McBain, Emma-Jayne Keevill, Catherine A. O’Neill

**Affiliations:** 1 Division of Musculoskeletal and Dermatological Sciences, Faculty of Biology, Medicine and Health, The University of Manchester, Manchester, United Kingdom; 2 Lydia Becker Institute of Immunology and Inflammation, Faculty of Biology, Medicine and Health, The University of Manchester, Manchester, United Kingdom; 3 Division of Pharmacy and Optometry, Faculty of Biology, Medicine and Health, The University of Manchester, Manchester, United Kingdom; 4 Biological Mass Spectrometry Facility, Faculty of Biology, Medicine and Health, The University of Manchester, Manchester, United Kingdom

**Keywords:** IL-33, keratinocyte, *Micrococcus luteus*, *Staphylococcus aureus*, TSLP, atopic dermatitis

## Abstract

*Staphylococcus aureus* second immunoglobulin-binding protein (Sbi) is a unique type 2-promoting virulence factor that induces IL-33 and thymic stromal lymphopoietin (TSLP) release. This mechanism is essential for the development of *S. aureus*–induced eczema in the widely used NC/Tnd mouse model of human atopic dermatitis (AD). Microbiome shifts in AD suggest that microbiota could modulate the disease. We therefore sought to identify skin bacteria that attenuate *S. aureus-*induced IL-33/TSLP release from keratinocytes. *Micrococcus luteus* was unique among skin isolates in its ability to negate cytokine induction. The bioactive factor responsible was identified using fractionation, LC-MS and recombinant proteins, as the serine protease “PA domain protein” (PADP). Immunoblotting and ELISA confirmed Sbi and IL-33 degradation by PADP. This was not observed with the *M. luteus* type strain which contains a frame shift mutation within the PADP active site. These data provide new insights into the role of skin microbiota in AD and highlights their potential as topical therapeutics.

## Introduction

Atopic dermatitis (AD) is the most common form of eczema and currently affects ~ 20% of children and 5% of adults in the western world (1, 2). The condition presents as chronic itch, dryness and erythema which can appear anywhere on the body (3). AD waxes and wanes and flares can be triggered by a multitude of environmental agents such as house dust mite allergen, pollens and very commonly, the skin bacterium *Staphylococcus aureus*. Indeed, approximately 90% of AD patients harbor *S. aureus* compared with ~10% of healthy individuals (4–6). The abundance of *S. aureus* increases greatly during AD flares, and the density of *S. aureus* is known to correlate with the severity of AD (7). To date, the literature suggests that there are no specific strains of *S. aureus* that are more associated with AD. However, some strains do show association with disease severity (8, 9).

Inflammation in AD is driven by type 2 cytokines, mainly IL-4 and IL-13 (10). The importance of type 2 cytokines is exemplified by a relatively new treatment for AD, dupilumab. This is an antibody that inhibits IL-4 and IL-13 signaling (11). Clinical trials have demonstrated the efficacy of dupilumab in improving moderate to severe AD but despite improvement at 4 months, biomarkers were not completely normalized (12). This suggests the involvement of triggering molecules upstream of IL-4 and IL-13.

IL-33 is upstream of type 2 cytokine induction from both innate and adaptive immune cells and is an important molecule bridging innate and adaptive responses in AD (13). Keratinocytes can initiate type 2 innate immune responses via secretion of IL-33 and a second pro type 2 cytokine, thymic stromal lymphopoietin (TSLP). Both IL-33 and TSLP are known to be elevated in the serum of AD patients compared to healthy individuals, and IL-33 levels correlate with disease severity (14, 15).

Until recently, IL-33 was thought to be an alarmin that is passively released from the nucleus of epidermal keratinocytes when the barrier is disrupted. However, we recently showed that *S. aureus* is an inducer of IL-33 and TSLP secretion from keratinocytes in the absence of cell death (16). We demonstrated that the secretome of *S. aureus*, which we term ‘FSA’ (Filtered supernatant of *S. aureus*), induces IL-33 and TSLP release from both primary keratinocytes and organ cultured human skin. Furthermore, this effect was specific to *S. aureus* and no other skin-associated staphylococci were able to elicit this response, whereas FSA from six different isolates of *S. aureus* induced IL-33 and TSLP. We showed that the NC/Tnd Japanese fancy-bred mouse, a model of human AD, develops the AD phenotype when exposed to FSA, and effect that could be abrogated using a neutralizing antibody to IL-33. These data directly link *S. aureus* to the type 2 phenotype in AD. We also showed that FSA contains a protein, the Second immunoglobulin binding protein (Sbi) which drives the release of IL-33 and TSLP. Thus, we proposed a model wherein the release of Sbi by *S. aureus* promotes the induction of IL-33 and TSLP which promote the type 2 response.

Suppression of *S. aureus* is a major treatment goal for AD therapies. Current treatment options include both oral and topical antibiotics for recurrent infections, but the repeated use of antibiotics increases the risk of antibiotic resistance (17). Hence, there is a significant need for more targeted therapies. Recent approaches have looked to skin microbiota for new therapeutic strategies (18).

Differences in microbiota composition have been reported for the skin of AD patients compared with healthy individuals, and there is increasing evidence that the microbiome can modulate the severity of inflammatory diseases (18–21). Studies investigating the therapeutic effect of commensal bacteria in AD inflammation show that selective killing of *S. aureus* has also been observed via the production of AMPs from *Staphylococcus epidermidis* and *Staphylococcus hominis* isolated from human skin (22). Unlike their presence in the normal population, strains possessing this antimicrobial activity were rarely found on AD patients, and low abundance correlated with skin colonization with *S. aureus.* Additionally, reintroduction of antimicrobial strains to human AD skin reduced colonization by *S. aureus*, demonstrating the therapeutic potential of topical bacteriotherapy in AD patients as an alternative to antibiotics which disrupt the microbiome through nonspecific killing (22).

As AD is associated with reduced microbial diversity and increased *S. aureus* presence, and *S. aureus* induces IL-33 and TSLP release which triggers AD pathogenesis, we hypothesized that specific members of the skin microbiota might mitigate AD pathogenesis by inhibiting *S. aureus*-induced IL-33 and TSLP release. Our aim, therefore, was to identify members of the skin microbiota able to attenuate *S. aureus-*induced IL-33 and TSLP release and to characterize the mechanisms involved.

## Methods

### Bacterial strains

*Micrococcus luteus* NCTC 2665 was purchased from the National Collection of Type Cultures (NCTC) (Public Health England, UK) and methicillin-sensitive *S. aureus* was isolated from a chronic foot wound in a diabetic patient as described previously in (23). Other bacteria mentioned, including *Micrococcus luteus* FAML, were isolated in a previous study from the skin of a healthy volunteer as described in (24). *M. luteus* FAML is banked under accession number (NCTC 23110401).

### Preparation of cell-free culture supernatants from bacteria

Single colonies of skin isolates were used to inoculate 5 ml Roswell Park Memorial Institute (RPMI) 1640 w/o: L-Glutamine, w/o: L-Tryptophan, w: 2.0 g/L NaHCO3, w/o Phenol Red (PAN Biotech UK Ltd, Dorset, UK) supplemented with L-Glutamine 200 mM (300 mg/L) (PAN Biotech UK Ltd) and 0.05% L-Tryptophan (Sigma-Aldrich). Cultures were incubated at 37˚C with shaking for 24 h unless stated otherwise. Samples were then centrifuged at 2706 x g for 10 min and filter sterilized using 0.22 µm syringe filters. In some experiments *M. luteus* FAML cell-free culture supernatant (CFCS) was pre-treated with 10 mM of the serine protease inhibitor 4-(2-Aminoethyl) benzenesulfonyl fluoride hydrochloride (AEBSF) (Sigma-Aldrich) for 1 h before use.

*S. aureus* FSA was prepared by inoculating nutrient broth with a single Microbank™ bead from a stock of methicillin-sensitive *S. aureus* followed by overnight incubation at 37˚C. A total of 10^8^ CFU were washed with Phosphate Buffered Saline (PBS) (Sigma-Aldrich) then resuspended in 10 ml Keratinocyte Growth Medium 2 (KGM) (PromoCell). Five ml were added to 15 ml fresh KGM and incubated for 6 h at 37˚C with shaking. After incubation, samples were centrifuged at 1600 x g for 5 min then filter sterilized using 0.22 µm filters (Starlab UK Ltd, England) and stored at –80˚C until required for stimulation experiments.

### Normal human epidermal keratinocyte culture and stimulation

Primary Normal Human Epidermal Keratinocytes (NHEK) (PromoCell, Heidelberg, Germany) were isolated from the epidermis of juvenile foreskin from pooled donors and grown in KGM supplemented with KGM SupplementMix (Promocell) at 37˚C in a humidified atmosphere of 5% CO_2_. NHEK were maintained in T75 tissue culture flasks and medium was replaced every 2 days to achieve ~90% confluency. Cells were cultured in 24-well tissue culture plates at a density of 5 x 10^4^ cells/well. Cells of passages 2 to 5 were used for experiments.

Once NHEK reached 70-90% confluence, medium was removed, and the cells were stimulated with 750 µl FSA to induce cytokine release. Stimulated cells were co-cultured with 250 µl of bacterial CFCS or bacterial culture medium control for up to 24 h at 37˚C in 5% CO_2_. NHEK culture medium was then removed and centrifuged at 10,000 x g for 15 min to remove cell debris before storing at -20˚C for downstream analysis.

### Human IL-33 and TSLP ELISA

To quantify IL-33 and TSLP in NHEK conditioned medium, human IL-33 (R&D systems) and human TSLP (R&D Systems) DuoSet ELISA Kits were used according to manufacturer’s instructions.

### Acetone precipitation of proteins

99.9% Acetone (Fisher Scientific) chilled to -20˚C was added to bacterial supernatant at a ratio of 4:1. The sample was then vortexed and incubated at -20˚C for 1 h. Following incubation, the sample was centrifuged for 10 min at 15,000 x g before decanting the supernatant and allowing the remaining acetone to evaporate. Protein precipitate was resuspended in either NHEK media or PBS to the required concentration.

### Immunoprecipitation of Sbi

Prior to immunoprecipitation, Sbi was concentrated by fractionating FSA using an Amicon Ultra-15–100 kDa cutoff membrane size exclusion centrifugal filter column (Merck Millipore, Germany) for 5 min at 2000 x g. The Dynabeads™ Protein G Immunoprecipitation Kit (Invitrogen, Loughborough, UK) was used to immunoprecipitate Sbi from FSA using IgG from human serum (Sigma-Aldrich) as per manufacturer’s instructions including antibody crosslinking.

### Immunoblot analysis

After gel electrophoresis using a NuPAGE™ 4-12% Bis-Tris gel (Invitrogen), a semi-wet transfer of proteins to a nitrocellulose membrane was carried out using the Bio-Rad Trans-Blot Turbo Transfer System (Bio-rad, California, USA) according to the manufacturer’s instructions. Immunoblotting was performed with rabbit anti-Sbi (1:1000) (gifted by Professor Jean Van Den Elsen, University of Bath), and horseradish peroxidase-conjugated goat anti-rabbit IgG (1:5000, BioRad, Hercules, USA). Protein bands were visualized in a ChemiDoc XRS+ System (Bio-rad) using enhanced chemiluminescence (ECL) reagent (GE Healthcare Amersham™, UK). All generated files were analyzed with Image Lab software (Bio-rad).

### Chromatography fractionation of the *M. luteus* FAML secretome

CFCS that had been previously concentrated by acetone precipitation was solubilized in PBS and separated by size exclusion chromatography (SEC) performed on a superdex200 (Cytiva) gel filtration column in 20mM TrisHCl with 150mM NaCl on a BioRad NGC chromatography system at a flow rate of 0.75ml/min, or by ion exchange chromatography (IEX) performed on a ResourceQ 5-ml ion exchange column loaded in 20mM TrisHCl in 50mM NaCl and eluted in a gradient to 0.5M NaCl. SEC and IEX was performed by The University of Manchester, Biomolecular Analysis Core Facility. Fractions were sterilized using 0.22 µm syringe filters before assessing their activity in FSA-stimulated NHEKs.

### Identification of potential active proteins using LC-MS

Fractions of *M. luteus* FAML secretome shown to have activity in FSA-stimulated NHEKs were added to 4x NuPAGE^®^ LDS sample buffer (Novex, Life Technologies), vortexed then heated at 70°C for 10 min before transferring to ice. Samples were loaded into a NuPAGE™ 10%, Bis-Tris gel and run into the top 5 mm for 5 min at 200V. The gel was stained with Coomassie blue then stored in dH2O at 4°C overnight.

In-gel digestion and tandem mass spectrometry were performed by The Biological Mass Spectrometry Facility at The University of Manchester (RRID code: SCR_020987) using an UltiMate^®^ 3000 Rapid Separation LC (RSLC, Dionex Corporation, Sunnyvale, CA) coupled to a QE HF (Thermo Fisher Scientific, Waltham, MA) mass spectrometer. Data produced were searched using Mascot (Matrix Science UK), against the [*Swi*ssprot and Trembl] database with taxonomy of Actinobacteria (which includes *M. luteus*) selected. Data were validated using Scaffold (Proteome Software, Portland, OR).

### Whole genome sequencing and variant calling

Whole genome sequencing was provided by MicrobesNG (http://www.microbesng.com). Pure cultures of each strain were grown in TSB to exponential phase. Cells were harvested and resuspended to the equivalent of 8–12 OD 600 nm in a tube containing 0.5 ml DNA/RNA Shield (Zymo Research, USA) following MicrobesNG strain submission procedures, and sent to MicrobesNG for sequencing. Libraries were sequenced on an lllumina NovaSeq 6000 (Illumina, San Diego, USA) using a 250 bp paired end protocol. *De novo* assembly was performed on samples using SPAdes version 3.7, and contigs annotated using Prokka 1.11. Variant calling against a selected isolate was performed using VarScan to identify genome mutations and single nucleotides polymorphisms (SNPs).

### Cloning and expression of PADP

The skin isolated *M. luteus* FAML PADP gene sequence was obtained using WGS. A 3kb fragment corresponding to the PADP gene was synthesized (Invitrogen, GeneArt) with flanking BamHI and Not1 restriction sites, subcloned into pET15 6His 3C vector and transferred to BL21 cells by the MRCPPU Reagents and Services (https://mrcppureagents.dundee.ac.uk [mrcppureagents.dundee.ac.uk]) at The University of Dundee. In some experiments, NHEKs were treated with 10 μg/ml of rPADP for 24 h.

### Statistical analysis

All experiments were carried out with three technical replicates and three or more biological replicates. Graphical data are presented as mean of biological replicates ± SEM using GraphPad Prism 10 software. Results were considered statistically significant if P-values were less than 0.05. All statistical tests were carried out using GraphPad Prism 10 software.

The Shapiro-Wilk test was used to determine whether a data set was normally distributed. Parametric single-factor data with two groups was analyzed using the unpaired *t* test. Single-factor data with three or more groups was tested using one-way ANOVA followed by multiple comparisons to a control group using Dunnett’s *post hoc* test. If normality could not be assumed, statistical comparisons between three or more groups were determined using the Kruskal-Wallis test with Dunn’s multiple comparisons.

## Results

### Cell-free culture supernatant from a *Micrococcus luteus* strain isolated from human skin negates *S. aureus-*induced release of IL-33 and TSLP in human keratinocytes

**Figure 1:**
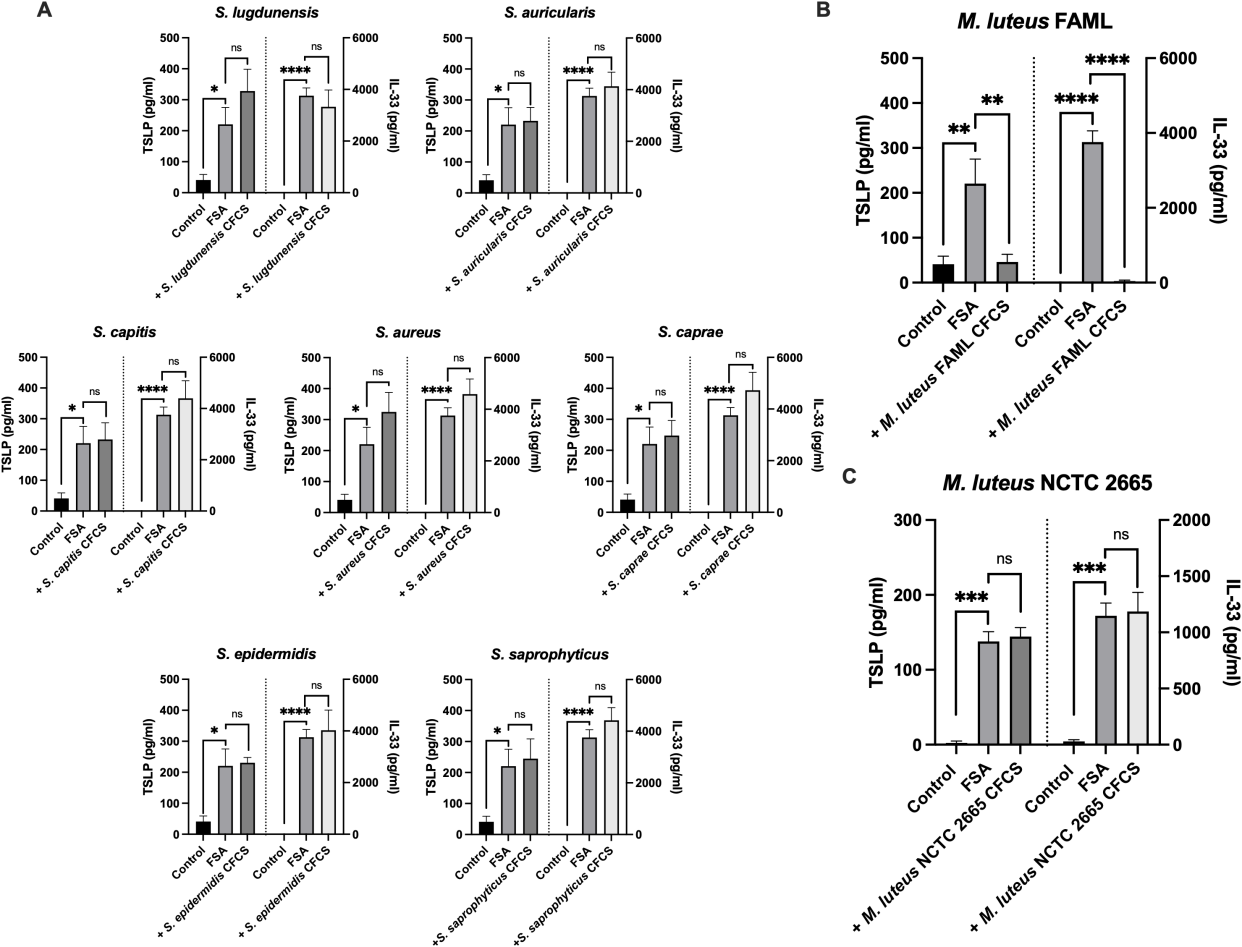
The ability to negate *S. aureus*-induced cytokine secretion from keratinocytes is a strain specific effect of *M. luteus* CFCS. **(A, B)** NHEK were treated with FSA or co-treated with FSA and skin bacterial CFCS for 24 h before quantifying IL-33 and TSLP in cell culture medium using ELISA. Stimulation of NHEK with FSA caused an increase in IL-33 and TSLP release. **(B)** Co-treatment with skin isolated *M. luteus* FAML CFCS negated FSA-induced release of IL-33 and TSLP. **(C)** Co-treatment with the *M. luteus* type strain NCTC 2665 had no effect on FSA-induced IL-33 and TSLP release. Data are expressed as mean ± SEM (n≥3). P values determined by one-way ANOVA *P ≤ 0.05 **P ≤ 0.01 ***P ≤ 0.001 ****P ≤ 0.0001 compared with FSA treated NHEK; ns, non-significant.

To identify members of the skin microbiota able to attenuate *S. aureus*-induced IL-33 and TSLP release, normal human epidermal keratinocytes (NHEK) were co-treated with filtered *S. aureus* supernatant (FSA) and cell-free culture supernatant (CFCS) from one of a panel of human skin isolates: *S. aureus, Micrococcus luteus, Staphylococcus auricularis, Staphylococcus lugdunensis, Staphylococcus epidermidis, Staphylococcus saprophyticus, Staphylococcus capitis* and *Staphylococcus caprae.* Over the time course of the experiment, none of the organisms produced CFCS that was toxic to NHEK as measured by MTT assay (**Supplementary Figure 1**). NHEK were incubated for 24 h with FSA and CFCS from skin isolates before quantifying IL-33 and TSLP in the cell culture medium by ELISA. In agreement with our previous study (16), the data in **Figure 1** demonstrate that treatment of NHEK with FSA causes an increase in IL-33 and TSLP release. No change in IL-33 or TSLP release was observed when NHEK were co-treated with FSA and CFCS from *S. aureus, S. auricularis, S. lugdunensis, S. epidermidis, S. saprophyticus, S. capitis* or *S. caprae* compared to FSA treatment alone (**Figure 1A**). However, co-treatment with CFCS from a skin isolate of *M. luteus (*which we term strain FAML) negated FSA-induced release of IL-33 and TSLP from NHEK (**Figure 1B**). This function of *M. luteus* FAML is not an attribute of all strains because the *M. luteus* type strain NCTC 2665 had no effect on FSA-induced released of IL-33 and TSLP in NHEK (**Figure 1C**). The efficacious molecule is produced early in the growth phase and is a putative protein.

**Figure 2:**
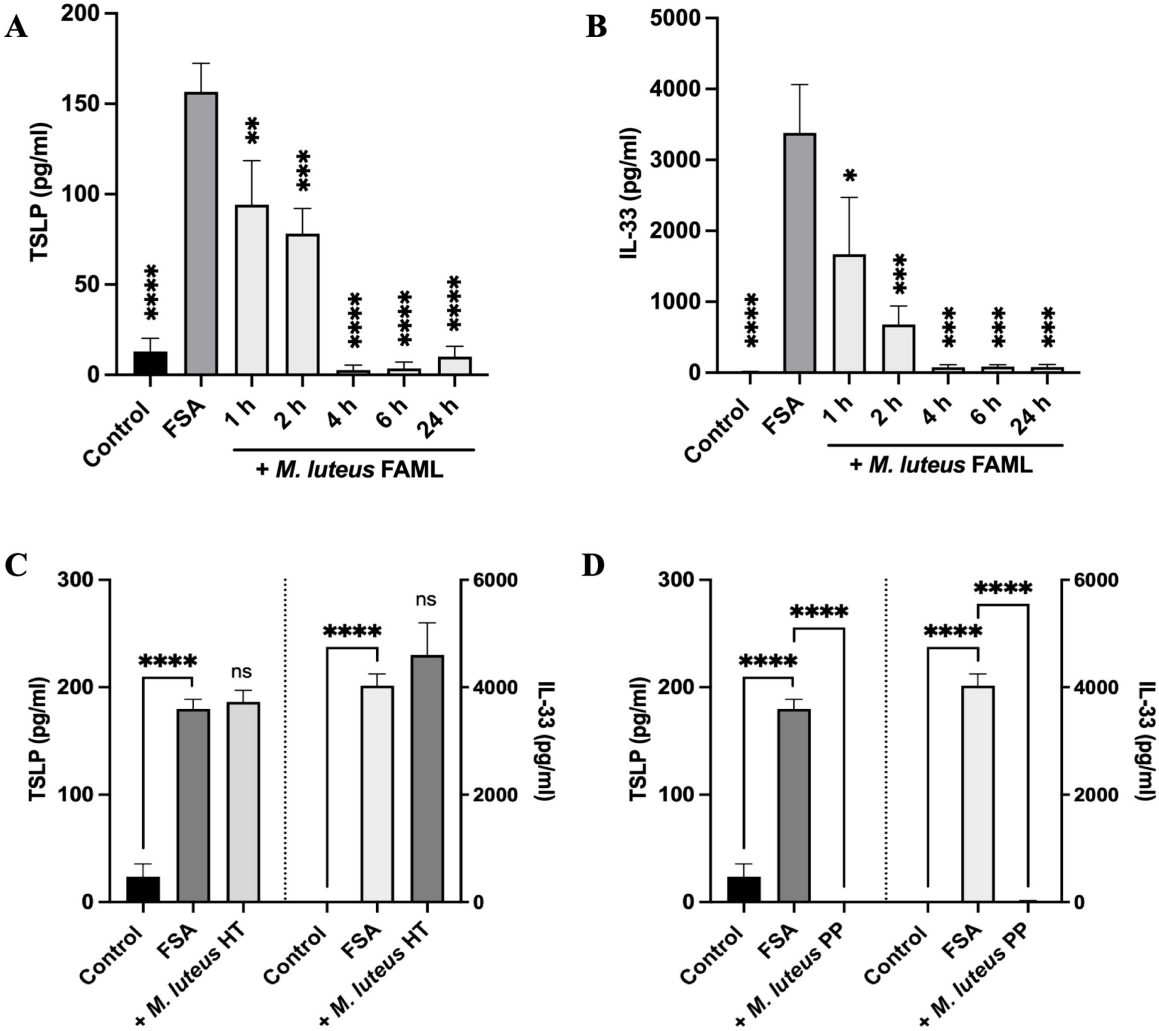
The efficacious molecule secreted by *M. luteus* FAML is a putative protein. *M. luteus* FAML was cultured for 1, 2, 4, 6 and 24 h before harvesting CFCS. NHEK were co-cultured with FSA and *M. luteus* FAML CFCS for 24 h before measuring **(A)** TSLP and **(B)** IL-33 in cell culture medium using ELISA. **(C)***M*. *luteus* FAML CFCS collected at 24 h lost activity against FSA-induced IL-33 and TSLP release in NHEK after heat treatment (HT) to 85°C. **(D)** Proteins within *M. luteus* FAML CFCS were precipitated using acetone, then reconstituted in cell culture medium before testing for activity using the same model. Activity was retained within the protein precipitate (PP). Data are expressed as mean ± SEM (n≥4). P values determined by one-way ANOVA *P ≤ 0.05 **P ≤ 0.01 ***P ≤ 0.001 ****P ≤ 0.0001 compared with FSA treated NHEK; ns, non-significant.

We next investigated the nature of the molecule(s) mediating the inhibitory effects on cytokine secretion. We determined that *M. luteus* FAML secretes the bioactive molecule as early as 1 h into the growth cycle (**Figures 2A, B**) whilst the bacterium is in lag phase (**Supplementary Figure 2**). *M. luteus* FAML CFCS collected after 24 h growth lost activity after heat treatment at 85°C (**Figure 2C**), whereas acetone precipitation of proteins within the CFCS retained activity (**Figure 2D**) suggesting that the bioactive molecule is a protein.

### *M. luteus* FAML negates FSA-induced IL-33 and TSLP release from NHEK via serine protease activity

**Figure 3:**
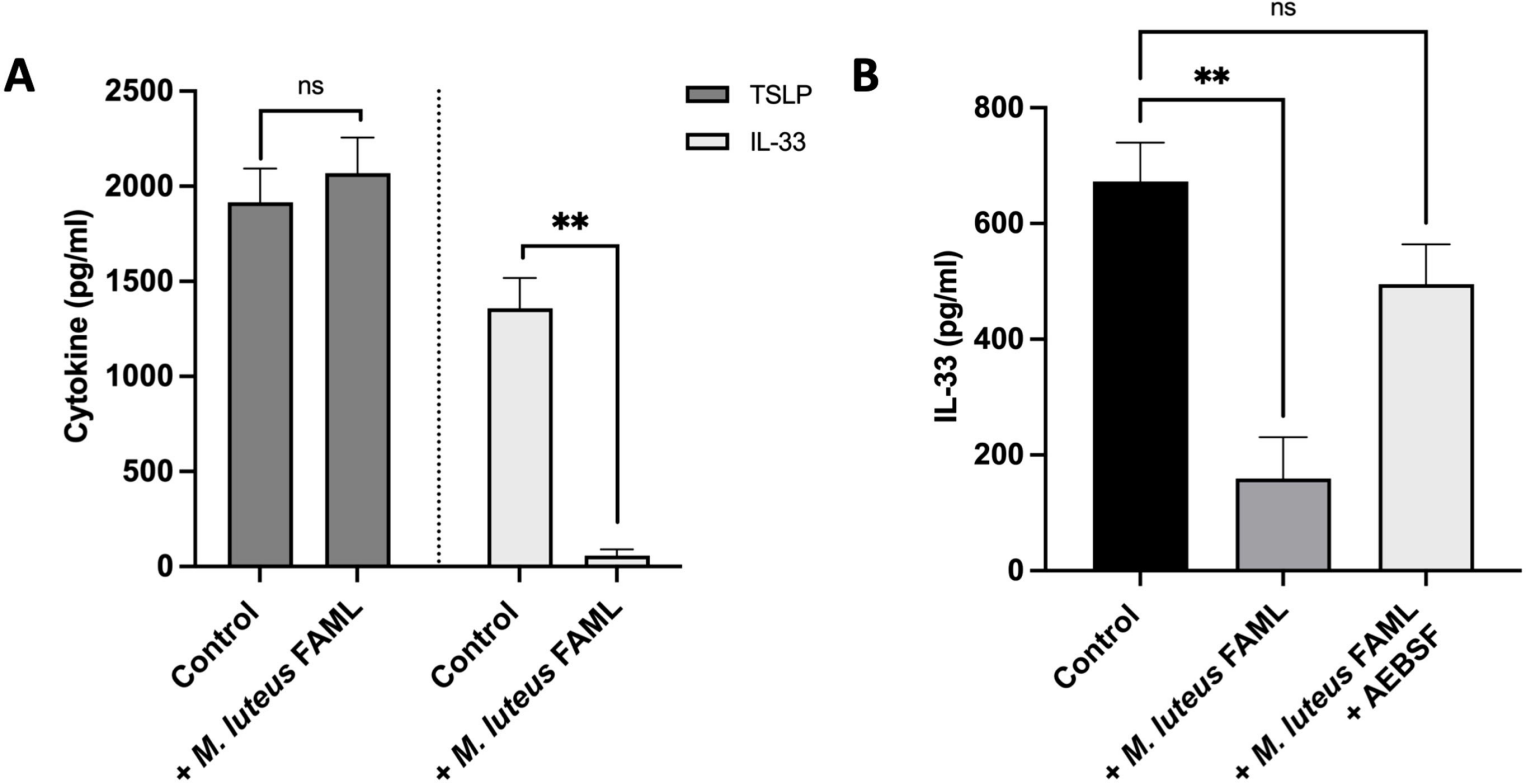
*M. luteus* FAML CFCS degrades IL-33 via serine protease activity. **(A)** Recombinant IL-33 or TSLP were treated with either *M. luteus* FAML CFCS or a medium control before quantifying the concentration of remaining IL-33 or TSLP within the sample using ELISA. Data are expressed as mean ± SEM (n=3). *M. luteus* FAML CFCS degraded IL-33 but not TSLP. P values determined by unpaired t test **P ≤ 0.01 compared with untreated IL-33 or TSLP control. **(B)***M. luteus* FAML CFCS was pre-treated with the serine protease inhibitor AEBSF. Recombinant IL-33 was then treated with either AEBSF treated *M. luteus* FAML CFCS or untreated *M. luteus* FAML CFCS. Pre-treatment of *M. luteus* FAML CFCS with AEBSF inhibited the anti-IL-33 activity. Data are expressed as mean ± SEM (n=3). P values determined by one-way ANOVA **P ≤ 0.01 compared with untreated IL-33 control; ns, non-significant.

To investigate the mechanism of the observed bioactivity, recombinant IL-33 and TSLP were independently treated with *M. luteus* FAML CFCS to investigate whether the reduction of IL-33 and TSLP in FSA-stimulated NHEK was due to a direct effect of the *M. luteus* FAML CFCS on these cytokines. The data in **Figure 3A** demonstrate the quantity of IL-33 and TSLP after treatment with *M. luteus* FAML CFCS compared to untreated controls as determined by ELISA. *M. luteus* FAML CFCS did not affect TSLP but significantly reduced the concentration of IL-33, which is indicative of protease activity.

To identify the mechanism of proteolysis and therefore understand more about the identity of the bioactive protein, the role of proteases in the degradation of IL-33 by *M. luteus* FAML CFCS was investigated. The most abundant proteolytic bacterial enzymes are serine proteases (25), and so *M. luteus* FAML CFCS was pre-treated with the irreversible serine protease inhibitor AEBSF before testing for activity. Recombinant IL-33 was treated with either AEBSF treated *M. luteus* FAML CFCS or untreated *M. luteus* FAML CFCS before quantifying the IL-33 concentration by ELISA. The data in **Figure 3B** demonstrates that the degradation of IL-33 treated with *M. luteus* FAML CFCS is inhibited by AEBSF.

### A serine protease of the *M. luteus* FAML secretome degrades *S. aureus* virulence factor Sbi in a dose dependent manner

**Figure 4:**
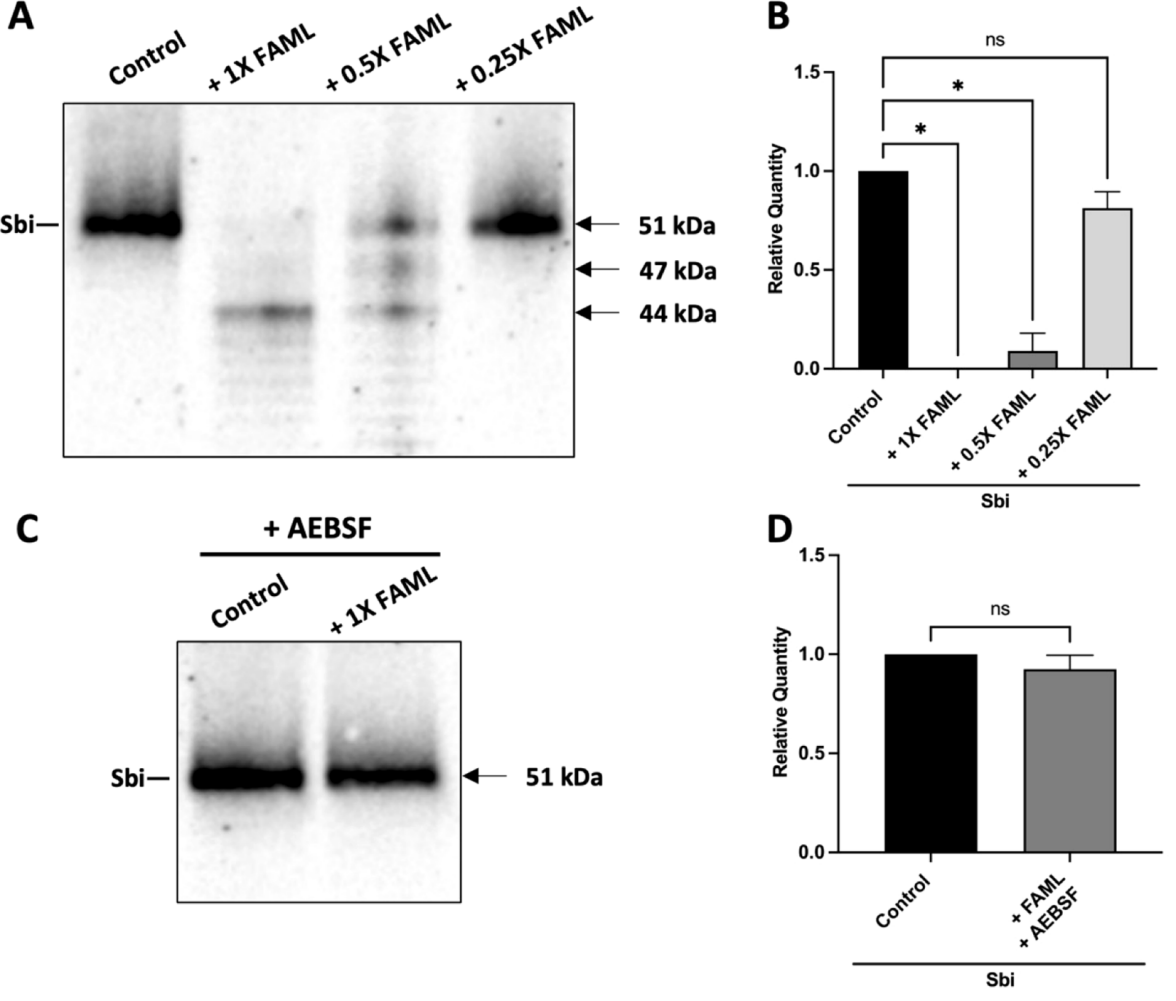
*M. luteus* FAML CFCS degrades Sbi in a dose dependent manner via serine protease activity. **(A)** Purified Sbi was treated for 1 h with either 1X, 0.5X or 0.25X concentration of *M. luteus* FAML CFCS before performing Western blot analysis. **(B)** The intensity of the Sbi band (51 kDa) was quantified using Image Lab and presented as relative quantity compared to the untreated control band. **(C)** Purified Sbi was treated for 1 h with AEBSF-treated *M. luteus* FAML CFCS. Sbi was also treated with AEBSF for 1 h as a control. **(D)** The intensity of the Sbi band was quantified using Image Lab and presented as relative quantity compared to the untreated control band. Western blot images **(A, C)** are representative of three and four individual experiments respectively. **(B, D)** Data are expressed as mean ± SEM (n≥ 3). P values determined by Kruskal-Wallis test *P ≤ 0.05 compared with the control; ns, non-significant.

Since *M. luteus* FAML CFCS degrades IL-33, but not TSLP, we considered that the mechanism involved in negation of TSLP release might involve proteolysis of its inducing factor, Sbi. Thus, Sbi was purified from FSA by immunoprecipitation using IgG, as confirmed by the presence of a 51 kDa band by Western blot (**Figure 4A**) (26). Purified Sbi was treated with different dilutions of *M. luteus* FAML CFCS for 1 h before performing Western blot analysis. The data in **Figures 4A, B** demonstrate that treatment of Sbi with *M. luteus* FAML CFCS resulted in degradation of Sbi in a dose-dependent manner, an effect that was inhibited by pre-treatment with AEBSF (**Figures 4C, D**).

### PA domain protein is present in all active *M. luteus* FAML CFCS fractions

To identify the bioactive molecule, *M. luteus* FAML CFCS concentrated by acetone precipitation was resolubilized and subjected to size exclusion chromatography (SEC) and ion exchange chromatography (IEX). Each fraction resulting from separation was tested for its ability to negate FSA-induced cytokine release. **Supplementary Table 1** lists all the proteins identified in each SEC fraction showing activity. **Table 1** shows the identity of the 10 proteins that were present in all four active SEC fractions. The proteins with the highest number of peptide matches across all samples and therefore the most certain identifications were “Protease associated” domain protein (PADP) (27), a member of the S8 family of serine proteases, and Glyceraldehyde-3-phosphate dehydrogenase (GAPDH). **Table 2** shows the identity of the eight proteins that were present in all three active IEX fractions analyzed (**Supplementary Table 2** lists all the proteins present in each of the three fractions showing activity). Other than GAPDH, PADP was the only protein identified in all active SEC fractions and IEX fractions.

**Table 1:** Proteins identified within all active SEC fractions by LC-MS analysis.

Identified proteins	SEC1	SEC2	SEC3	SEC4
PA domain protein OS=*Micrococcus luteus* SK58	11	8	3	1
Glyceraldehyde-3-phosphate dehydrogenase OS=*Micrococcus luteus* SK58	8	7	1	2
Uncharacterized protein OS=*Micrococcus luteus* SK58	2	2	2	2
DivIVA domain-containing protein OS=*Rhodococcus qingshengii*	2	2	1	2
Hydrolase, alpha/beta domain protein OS=*Micrococcus luteus*	3	2	1	1
Uncharacterized protein OS=*Micrococcus luteus*	4	1	1	1
ATP synthase subunit alpha OS=*Rhodococcus aetherivorans*	3	1	1	2
Phosphatase OS=*Rhodococcus qingshengii*	3	1	1	1
Type III secretion system (T3SS) inner membrane Yop/YscD-like protein OS=*Micrococcaceae bacterium* JKS001869	3	1	2	1
Mycolyltransferase OS=*Rhodococcus qingshengii*	2	1	1	1

List of proteins that were identified in all four active SEC fractions analyzed. The numbers in the columns represent the number of unique peptides that have been matched to the identified protein in that sample. The greater the number of matches the more certain the identification; 1 match is a possible identification, 2–3 matches a probable identification, 4 and over an almost certain identification. OS, Origin species.

**Table 2:** Proteins identified within all active IEX fractions by LC-MS analysis.

Identified proteins	IEX1	IEX2	IEX3
Uncharacterized protein (Fragment) OS=*Microbacterium* sp. AISO3	3	1	16
Glyceraldehyde-3-phosphate dehydrogenase OS=*Micrococcus luteus*	11	7	9
Catalase OS=*Micrococcus luteus*	7	4	14
Catalase OS=*Micrococcus* sp. HMSC31B01	6	4	13
**PA domain protein OS=*Micrococcus luteus***	**6**	**6**	**2**
Phosphate-binding protein PstS OS=*Micrococcus luteus* Mu201	3	3	3
Dihydrolipoyl dehydrogenase OS=*Micrococcus luteus*	4	2	4
Putative phenylalanine aminotransferase OS=*Micrococcus luteus*	3	2	3

List of proteins that were identified in all three active IEX fractions analyzed. The numbers in the columns represent the number of unique peptides that have been matched to the identified protein in that sample. The greater the number of matches the more certain the identification; 1 match is a possible identification, 2–3 matches a probable identification, 4 and over an almost certain identification. OS, Origin species.

The text in bold denotes the active protein responsible for the observed effects.

### Recombinant PADP recapitulates the effects of *M. luteus* FAML CFCS

**Figure 5:**
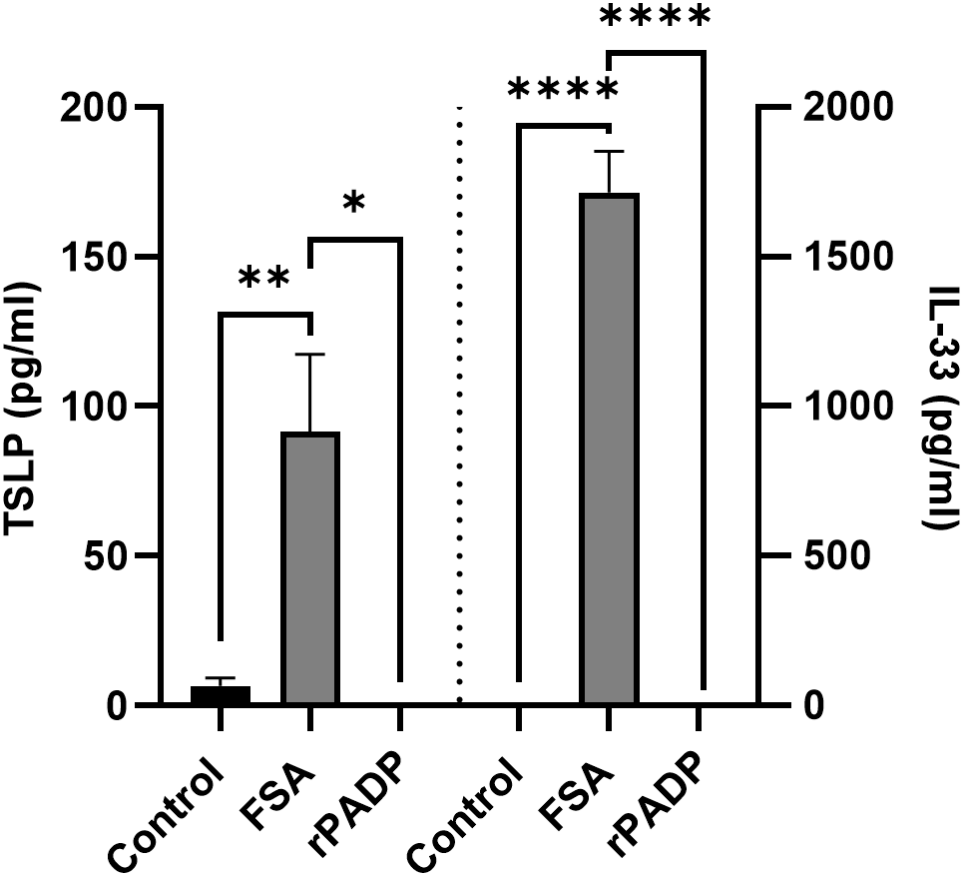
Recombinant PADP negates FSA-induced IL-33 and TSLP release from NHEK. NHEK were co-cultured with FSA and rPADP for 24 h before measuring IL-33 and TSLP in the cell culture medium using ELISA. The rPADP negated FSA-induced IL-33 and TSLP release in NHEK. Data are expressed as mean ± SEM (n≥3). P values determined by one-way ANOVA *P ≤ 0.05 **P ≤ 0.01 ****P ≤ 0.0001 compared with FSA treated NHEK.

To confirm PADP as the active molecule within *M. luteus* FAML CFCS, a recombinant PADP (rPADP) encoded by the genome sequence from *M. luteus* FAML as determined by whole genome sequencing (WGS) (**Supplementary Figure 3**) was produced by cloning and expression in an *Escherichia coli* vector system. The rPADP was tested for activity against FSA-induced release of IL-33 and TSLP from NHEK. The rPADP negated FSA-induced release of IL-33 and TSLP from NHEK (**Figure 5**).

**Figure 6:**
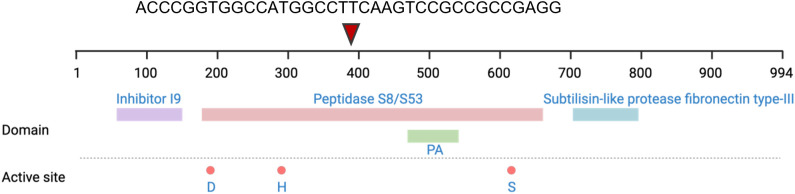
A representative diagram to show the location of each domain and active site within the *M. luteus* FAML PADP sequence. In the *M. luteus* NCTC 2665 sequence a frame shift insertion occurs at amino acid 390, splitting the peptidase domain, producing a truncated protein and a loss of function. D, Aspartate; H, Histidine; S, Serine.

To further implicate PADP as the protein responsible for *M. luteus* FAML CFCS activity, WGS and variant calling analysis was performed for the bioactive skin isolated *M. luteus* FAML and the inactive type strain *M. luteus* NCTC 2665 to investigate differences in the genome sequence encoding the PADP. The NCTC 2665 type strain genome sequence was aligned to the skin isolated *M. luteus* FAML genome sequence as a reference to identify variations by bioinformatics analysis. Multiple non-synonymous coding mutations were identified in the *M. luteus* NCTC 2665 type strain PADP encoding gene, as well as a frame shift insertion mutation occurring at amino acid 390 in the sequence, which is predicted to result in a truncated protein. The PA protein peptidase S8/S53 domain occurs from amino acid 160-648, and the aspartic acid, histidine and serine residues that comprise the catalytic triad occur at position 195, 292 and 615 respectively (**Figure 6**) (27, 28). Consequently, the protein truncation observed in the *M. luteus* type strain would split the peptidase S8/S53 domain and result in the loss of the catalytic triad. The frame shift and non-synonymous coding mutations and the resulting amino acid substitutions are listed in **Supplementary Table 3.**

## Discussion

Current treatment options for AD are limited and often focus on management of symptoms through elimination of *S. aureus*. Due to the increased prevalence of antibiotic resistance, interest has turned to the development of alternative strategies to treat *S. aureus* infection. One such approach is the targeting of virulence factors.

Sbi is a *S. aureus* virulence factor, which contributes to immune evasion and the induction of the host inflammatory response to the bacterium (16, 26, 29). We previously showed that Sbi induces IL-33 and TSLP release from keratinocytes and *ex vivo* skin. It is these two cytokines that promote the inflammatory cascade characteristic of AD. The data from the present study suggest that this cascade could be ‘short circuited’ by a member of the healthy skin microbiome. The strain of *M. luteus* we have isolated from healthy human skin inhibits the induction of IL-33 and TSLP in response to *S. aureus in vitro*. To the best of our knowledge this is the first report of a skin-isolated bacterium preventing a *S. aureus-*induced type 2 cytokine release from keratinocytes. If these novel results were recapitulated *in vivo*, PA domain protease could be used to interfere with Sbi to prevent stimulation of the inflammatory response. Therapeutic blockade of IL-33 (etokimab) and TSLP (tezepelumab) has not yielded substantial clinical benefit in AD (30). However, both drugs inhibit these cytokines only after their release, at a point when downstream cytokine cascades may already be established. In contrast, PA domain protease from *M. luteus* FAML presents a novel upstream approach by degrading, Sbi, the initiator of IL-33 and TSLP release. By preventing the induction of these cytokines in the first place, this approach could avert downstream inflammatory cascades and translate into clinical benefit.

The mechanism underpinning negation of IL-33 and TSLP is likely to be proteolysis of Sbi by the secreted serine protease PADP. This is evidenced in several ways: firstly, *M. luteus* FAML secretome contains a proteolytic activity that degrades Sbi. Secondly, PADP is present in all fractions displaying activity when *M. luteus* FAML CFCS is separated chromatographically. Thirdly, recombinant PADP shows a similar ability to negate Il-33/TSLP induction as the *M. luteus* FAML CFCS. Finally, strains of *M. luteus* with an inactive PADP have no ability to inhibit induction of Il-33 and TSLP.

Degradation of IL-33 is evidenced by the loss of IL-33 when treated directly with the *M. luteus* FAML CFCS. There are a limited number of reports of degradation of cytokines via bacterial proteases (31–33). However, to the best of our knowledge, this is the first report of bacterial degradation of IL-33. It is likely that the primary cause for the negation of IL-33 in FSA-stimulated NHEK co-treated with *M. luteus* FAML CFCS is the degradation of Sbi preventing the induction of IL-33 release. However, it is conceivable that any IL-33 released would itself be degraded by protease activity. Considering a lack of any direct proteolytic effect on TSLP, this also presents further evidence that the most plausible explanation for *M. luteus* FAML CFCS treatment negating TSLP release is proteolysis of Sbi.

Interestingly, the observed activity of PADP seems to be unique to *M. luteus* FAML, as other common skin isolated species tested had no activity against FSA-induced IL-33 and TSLP release in NHEK. Furthermore, the ability to inhibit IL-33/TSLP induction may be strain specific because the *M. luteus* type strain NCTC 2665 was inactive. WGS and variant calling analysis revealed a frame shift mutation within the ineffective *M. luteus* NCTC 2665 PADP active site compared to the effective *M. luteus* FAML, providing a possible explanation as to why this particular strain was ineffective.

In contrast to observations in severe AD lesions, a recent study exploring the composition of the skin microbiome in mild AD reported that the abundance of *S. aureus* did not differ between mild AD lesions and healthy skin (34). In fact, mild AD lesions were characterized by an increased abundance of other non-staphylococcus species and a reduced abundance of species of 15 genera. Interestingly, the species with the third highest fold decrease was *M. luteus* (5.3-fold), suggesting that this bacterium could have a potentially protective function that is depleted in mild AD (34). Smith et al. demonstrated that all 16 clinical *S. aureus* isolates tested expressed Sbi in either the culture supernatant, the cytoplasmic membrane or both (26). Since production of Sbi appears to be common amongst *S. aureus* strains, we hypothesize that the depletion of PADP-producing *M. luteus* on the skin results in reduced degradation of Sbi and consequently higher levels on the skin. This could potentially stimulate the type 2 immune response even when the abundance of *S. aureus* is similar to that of healthy skin.

The observation that *M. luteus* FAML secretes a serine protease which degrades Sbi and therefore inhibits the induction of IL-33 and TSLP in NHEK provides preliminary evidence of the ability of resident skin bacteria to interfere with pathogen virulence factors in order to mitigate an AD-like immune response. The importance of the resident skin commensal bacteria in interfering with *S. aureus* colonization of the skin is an emerging theme. For example, coagulase negative staphylococci such as *S. hominis* A9 are able to produce peptide antibiotics (termed lantibiotics) that can inhibit cell wall biosynthesis of gram-positive bacteria including *S. aureus* (18). Similarly, the fibupeptide, lugdunin, produced by *S. lugdunensis* possesses potent anti-*S. aureus* activity. Indeed, humans who are colonized by *S. lugdunensis* have a six-fold lower carriage of *S. aureus* in the nose than non-colonized individuals (35). There are also a number of enzymes and metabolites produced by skin commensals that can interfere with *S. aureus* functions such as adhesion and quorum sensing [reviewed in (36)]. The degradation of both Sbi and IL-33 by PADP presents a possible new mechanism that could be exploited to prevent *S. aureus* skin infections and contribute to the development of novel AD therapeutics. Overall, these results advance the understanding of microbe-microbe interactions on the skin and their implications for inflammatory skin disorders.

## Data Availability

The original contributions presented in the study are included in the article/**Supplementary Material**. Further inquiries can be directed to the corresponding author.

## References

[1] Barbarot S Auziere S Gadkari A Girolomoni G Puig L Simpson EL . Epidemiology of atopic dermatitis in adults: Results from an international survey. Allergy. (2018) 73:1284–93. doi: 10.1111/all.2018.73.issue-6 29319189

[2] Kowalska-Olędzka E Czarnecka M Baran A . Epidemiology of atopic dermatitis in Europe. J Drug Assess. (2019) 8:126–8. doi: 10.1080/21556660.2019.1619570, PMID: 31232396 PMC6566979

[3] Kapur S Watson W Carr S . Atopic dermatitis. Allergy Asthma Clin Immunol. (2018) 14:52. doi: 10.1186/s13223-018-0281-6, PMID: 30275844 PMC6157251

[4] Kong HH Oh J Deming C Conlan S Grice EA Beatson MA . Temporal shifts in the skin microbiome associated with disease flares and treatment in children with atopic dermatitis. Genome Res. (2012) 22:850–9. doi: 10.1101/gr.131029.111, PMID: 22310478 PMC3337431

[5] Gonzalez ME Schaffer JV Orlow SJ Gao Z Li H Alekseyenko AV . Cutaneous microbiome effects of fluticasone propionate cream and adjunctive bleach baths in childhood atopic dermatitis. J Am Acad Dermatol. (2016) 75:481–93. doi: 10.1016/j.jaad.2016.04.066, PMID: 27543211 PMC4992571

[6] Byrd AL Deming C Cassidy SKB Harrison OJ Ng WI Conlan S . Staphylococcus aureus and Staphylococcus epidermidis strain diversity underlying pediatric atopic dermatitis. Sci Transl Med. (2017). 9:eaal4651. doi: 10.1126/scitranslmed.aal4651, PMID: 28679656 PMC5706545

[7] Totté JEE van der Feltz WT Hennekam M van Belkum A van Zuuren EJ Pasmans SGMA . Prevalence and odds of Staphylococcus aureus carriage in atopic dermatitis: a systematic review and meta-analysis. Br J Dermatol. (2016) 175:687–95., PMID: 26994362 10.1111/bjd.14566

[8] Blicharz L Szymanek-Majchrzak K Młynarczyk G Czuwara J Waśkiel-Burnat A Goldust M . Multilocus-sequence typing reveals clonality of Staphylococcus aureus in atopic dermatitis. Clin Exp Dermatol. (2023) 48:1341–6. doi: 10.1093/ced/llad262, PMID: 37566920

[9] Benito D Aspiroz C Gilaberte Y Sanmartín R Hernández-Martin Á Alonso M . Genetic lineages and antimicrobial resistance genotypes in Staphylococcus aureus from children with atopic dermatitis: detection of clonal complexes CC1, CC97 and CC398. J Chemother. (2016) 28:359–66. doi: 10.1179/1973947815Y.0000000044, PMID: 26027683

[10] Suárez-Fariñas M Tintle SJ Shemer A Chiricozzi A Nograles K Cardinale I . Nonlesional atopic dermatitis skin is characterized by broad terminal differentiation defects and variable immune abnormalities. J Allergy Clin Immunol. (2011) 127:954–964.e4. doi: 10.1016/j.jaci.2010.12.1124, PMID: 21388663 PMC3128983

[11] Gandhi NA Bennett BL Graham NMH Pirozzi G Stahl N Yancopoulos GD . Targeting key proximal drivers of type 2 inflammation in disease. Nat Rev Drug Discov. (2016) 15:35–50. doi: 10.1038/nrd4624, PMID: 26471366

[12] Guttman-Yassky E Bissonnette R Ungar B Suárez-Fariñas M Ardeleanu M Esaki H . Dupilumab progressively improves systemic and cutaneous abnormalities in patients with atopic dermatitis. J Allergy Clin Immunol. (2019) 143:155–72. doi: 10.1016/j.jaci.2018.08.022, PMID: 30194992

[13] Pinto SM Subbannayya Y Rex DAB Raju R Chatterjee O Advani J . A network map of IL-33 signaling pathway. J Cell Commun Signal. (2018) 12:615–24. doi: 10.1007/s12079-018-0464-4, PMID: 29705949 PMC6039344

[14] Nygaard U Hvid M Johansen C Buchner M Fölster-Holst R Deleuran M . TSLP, IL-31, IL-33 and sST2 are new biomarkers in endophenotypic profiling of adult and childhood atopic dermatitis. J Eur Acad Dermatol Venereol. (2016) 30:1930–8. doi: 10.1111/jdv.2016.30.issue-11, PMID: 27152943

[15] Tamagawa-Mineoka R Okuzawa Y Masuda K Katoh N . Increased serum levels of interleukin 33 in patients with atopic dermatitis. J Am Acad Dermatol. (2014) 70:882–8. doi: 10.1016/j.jaad.2014.01.867, PMID: 24569114

[16] Al Kindi A Williams H Matsuda K Alkahtani AM Saville C Bennett H . Staphylococcus aureus second immunoglobulin-binding protein drives atopic dermatitis via IL-33. J Allergy Clin Immunol. (2021) 147:1354–1368.e3. doi: 10.1016/j.jaci.2020.09.023, PMID: 33011245

[17] Miller LG Eells SJ David MZ Ortiz N Taylor AR Kumar N . Staphylococcus aureus skin infection recurrences among household members: An examination of host, behavioral, and pathogen-level predictors. Clin Infect Dis. (2015). 60:753–63. doi: 10.1093/cid/ciu943, PMID: 25428411 PMC4402346

[18] Nakatsuji T Hata TR Tong Y Cheng JY Shafiq F Butcher AM . Development of a human skin commensal microbe for bacteriotherapy of atopic dermatitis and use in a phase 1 randomized clinical trial. Nat Med. (2021) 27:700–9. doi: 10.1038/s41591-021-01256-2, PMID: 33619370 PMC8052297

[19] Lebeer S Oerlemans E Claes I Wuyts S Henkens T Spacova I . Topical cream with live lactobacilli modulates the skin microbiome and reduce acne symptoms. bioRxiv. (2018).

[20] Myles IA Earland NJ Anderson ED Moore IN Kieh MD Williams KW . First-in-human topical microbiome transplantation with Roseomonas mucosa for atopic dermatitis. JCI Insight. (2018) 3. doi: 10.1172/jci.insight.120608, PMID: 29720571 PMC6012572

[21] Chen D He J Li J Zou Q Si J Guo Y . Microbiome and metabolome analyses reveal novel interplay between the skin microbiota and plasma metabolites in psoriasis. Front Microbiol. (2021) 12:643449. doi: 10.3389/fmicb.2021.643449, PMID: 33796091 PMC8007969

[22] Nakatsuji T Chen TH Narala S Chun KA Two AM Yun T . Antimicrobials from human skin commensal bacteria protect against Staphylococcus aureus and are deficient in atopic dermatitis. Sci Transl Med. (2017) 9:4680. doi: 10.1126/scitranslmed.aah4680, PMID: 28228596 PMC5600545

[23] Oates A Bowling FL Boulton AJM McBaina AJ . Molecular and culture-based assessment of the microbial diversity of diabetic chronic foot wounds and contralateral skin sites. J Clin Microbiol. (2012) 50:2263–71. doi: 10.1128/JCM.06599-11, PMID: 22553231 PMC3405613

[24] Elias AE McBain AJ Aldehalan FA Taylor G O’Neill CA . Activation of the aryl hydrocarbon receptor via indole derivatives is a common feature in skin bacterial isolates. J Appl Microbiol. (2024) 135. doi: 10.1093/jambio/lxae273, PMID: 39444068

[25] Caminero A Guzman M Libertucci J Lomax AE . The emerging roles of bacterial proteases in intestinal diseases. Gut Microbes. (2023) 15. doi: 10.1080/19490976.2023.2181922, PMID: 36843008 PMC9980614

[26] Smith EJ Visai L Kerrigan SW Speziale P Foster TJ . The sbi protein is a multifunctional immune evasion factor of staphylococcus aureus. Infect Immun. (2011) 79:3801. doi: 10.1128/IAI.05075-11, PMID: 21708997 PMC3165492

[27] Madupu R Durkin AS Torralba M Methe B Sutton GG Strausberg RL . PA domain protein [Micrococcus luteus SK58] - Protein. Rockville, MD, USA: NCBI. (2009).

[28] UniProt . (2024). Available online at: C5C8L7_MICLC/Subtilasefamilyprotease/peptidase inhibitor I9 (Accessed December 09, 2024).

[29] Gonzalez CD Ledo C Giai C Garófalo A Gómez MI . The Sbi Protein Contributes to Staphylococcus aureus Inflammatory Response during Systemic Infection. PloS One. (2015) 10:e0131879. doi: 10.1371/journal.pone.0131879, PMID: 26126119 PMC4488394

[30] Uppal SK Kearns DG Chat VS Han G Wu JJ . Review and analysis of biologic therapies currently in phase II and phase III clinical trials for atopic dermatitis. J Dermatol Treat. (2022) 33:626–36. doi: 10.1080/09546634.2020.1775775, PMID: 32507066

[31] Theander TG Kharazmi A Pedersen BK Christensen LD Tvede N Poulsen LK . Inhibition of human lymphocyte proliferation and cleavage of interleukin-2 by Pseudomonas aeruginosa proteases. Infect Immun. (1988) 56:1673–7. doi: 10.1128/iai.56.7.1673-1677.1988, PMID: 3133317 PMC259461

[32] Fletcher J Reddi K Poole S Nair S Henderson B Tabona P . Interactions between periodontopathogenic bacteria and cytokines. J Periodontal Res. (1997) 32:200–5. doi: 10.1111/j.1600-0765.1997.tb01406.x, PMID: 9085235

[33] Parmely M Gale A Clabaugh M Horvat R Zhou W-W . Proteolytic inactivation of cytokines by pseudomonas aeruginosa. Infect Immun. (1990) 58:3009–14. doi: 10.1128/iai.58.9.3009-3014.1990, PMID: 2117578 PMC313603

[34] Delanghe L De Boeck I Van Malderen J Allonsius CN Van Rillaer T Bron PA . Mild atopic dermatitis is characterized by increase in non-staphylococcus pathobionts and loss of specific species. Sci Rep. (2024) 14:23659. doi: 10.1038/s41598-024-74513-2, PMID: 39390034 PMC11467409

[35] Zipperer A Konnerth MC Laux C Berscheid A Janek D Weidenmaier C . Human commensals producing a novel antibiotic impair pathogen colonization. Nature. (2016) 535:511–6. doi: 10.1038/nature18634, PMID: 27466123

[36] Bier K Schittek B . Beneficial effects of coagulase-negative Staphylococci on Staphylococcus aureus skin colonization. Exp Dermatol. (2021) 30:1442–52. doi: 10.1111/exd.v30.10, PMID: 33960019

[37] Elias A . Anti-inflammatory skin microbiota and their potential as topical therapeutics. Manchester, UK: University of Manchester (2024).

