# Supplementary material for: A skin isolate of Micrococcus luteus negates the Staphylococcus aureus-induced release of type 2 cytokines from keratinocytes
Source: Front Immunol. 2026 Jan 28;17:1711723. doi: 10.3389/fimmu.2026.1711723 (PMC12891184; doi:10.3389/fimmu.2026.1711723)
Supplement: Supplementary file 1 [file Table1.docx]

**Supplementary figures**

Supplementary Figure 1 Viability of NHEK treated with CFCS from skin isolates.

Skin isolates were grown for 96 h before collecting CFCS. NHEK were treated with skin isolate CFCS for 24 h before measuring cell viability using MTT assay. Results are expressed relative to the untreated control. Data are expressed as mean ± SEM (n≥3). Significance was determined using the Kruskal-Wallis test with Dunn’s multiple comparisons with the control.


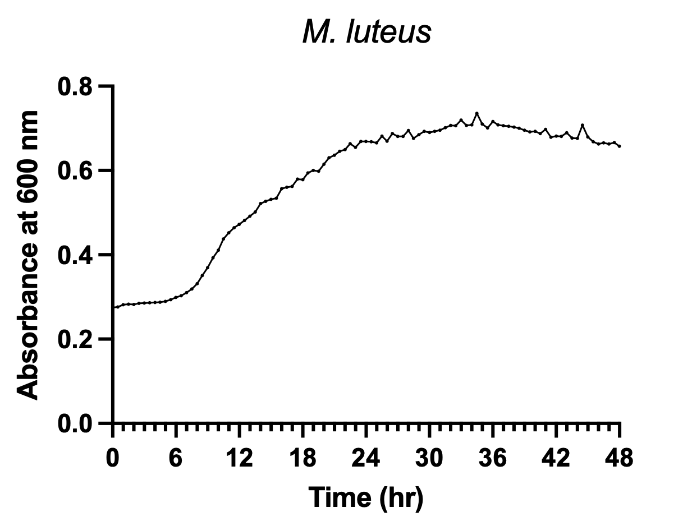


Supplementary Figure 2 The growth curve of *M. luteus* FAML isolated from human skin.

*M. luteus* FAML is in lag phase until ~ 7 h post inoculation. Data are presented as mean (n=3).

**Supplementary Table 1 All proteins identified within active SEC fractions by LC-MS analysis.** Highlighted proteins are present in all four SEC fractions analysed. The numbers in the columns represent the number of unique peptides that have been matched to the identified protein in that sample. The greater the number of matches the more certain the identification; 1 match is a possible identification, 2 – 3 matches a probable identification, 4 and over an almost certain identification. Origin species (OS).

| **Identified Proteins (51)** | **SEC**  **1** | **SEC**  **2** | **SEC**  **3** | **SEC**  **4** |
| --- | --- | --- | --- | --- |
|  |  |  |  |  |
| PA domain protein OS=*Micrococcus luteus* SK58 | 11 | 8 | 3 | 1 |
| Ferredoxin OS=*Micrococcus luteus* | 2 | 2 |  |  |
| Uncharacterized protein OS=*Micrococcus luteus* SK58 | 5 | 4 |  |  |
| Dihydrolipoyl dehydrogenase OS=*Micrococcus luteus* SK58 | 11 | 11 |  |  |
| Deoxyribose-phosphate aldolase OS=*Micrococcus luteus* | 7 | 6 | 1 |  |
| Glyceraldehyde-3-phosphate dehydrogenase OS=*Micrococcus* *luteus* SK58 | 8 | 7 | 1 | 2 |
| ABC transporter, substrate-binding protein, family 3 OS=*Micrococcus luteus* SK58 | 8 | 7 |  |  |
| Uncharacterized protein OS=*Micrococcus luteus* SK58 | 2 | 2 | 2 | 2 |
| Major royal jelly protein OS=*Micrococcus luteus* SK58 | 6 | 8 |  |  |
| Uncharacterized protein OS=*Micrococcus luteus* SK58 | 7 | 7 |  |  |
| Uncharacterized protein (Fragment) OS=*Microbacterium* *sp*. AISO3 | 8 |  | 1 |  |
| Phosphate-binding protein PstS OS=*Micrococcus luteus* Mu201 | 10 | 4 |  |  |
| Putative phenylalanine aminotransferase OS=*Micrococcus luteus* | 4 | 7 |  |  |
| Indole-3-glycerol phosphate synthase OS=*Micrococcus luteus* | 5 | 4 | 1 |  |
| Peptide/nickel transport system substrate-binding protein OS=*Micrococcaceae* *bacterium* JKS001869 | 5 | 5 |  |  |
| Glyceraldehyde-3-phosphate dehydrogenase OS=*Rhodococcus erythropolis* (strain PR4 / NBRC 100887) | 4 |  | 1 | 3 |
| Catalase OS=*Micrococcus luteus* | 4 | 4 |  |  |
| Imidazoleglycerol-phosphate dehydratase OS=*Micrococcus luteus* | 3 | 5 |  |  |
| Phosphoglycerate kinase OS=*Micrococcus luteus* SK58 | 3 | 4 |  |  |
| DivIVA domain-containing protein OS=*Rhodococcus qingshengii* | 2 | 2 | 1 | 2 |
| Uncharacterized protein OS=*Rhodococcus enclensis* | 3 | 2 |  | 1 |
| Hydrolase, alpha/beta domain protein OS=*Micrococcus luteus* | 3 | 2 | 1 | 1 |
| Uncharacterized protein OS=*Micrococcus luteus* | 4 | 1 | 1 | 1 |
| Transaldolase OS=*Micrococcus luteus* | 5 | 4 |  |  |
| ATP synthase subunit alpha OS=*Rhodococcus aetherivorans* | 3 | 1 | 1 | 2 |
| Phosphatase OS=*Rhodococcus qingshengii* | 3 | 1 | 1 | 1 |
| Type III secretion system (T3SS) inner membrane Yop/YscD-like protein OS=*Micrococcaceae bacterium* JKS001869 | 3 | 1 | 2 | 1 |
| Lipoprotein OS=*Rhodococcus qingshengii* | 4 | 1 |  | 2 |
| Thioesterase OS=*Micrococcus* *sp*. CH7 | 3 | 2 |  |  |
| Aminotransferase OS=*Micrococcus luteus* | 3 | 3 |  |  |
| Chlorite O(2)-lyase OS=*Micrococcus luteus* | 3 | 4 |  |  |
| 2,3,4,5-tetrahydropyridine-2,6-dicarboxylate N-acetyltransferase OS=*Micrococcus luteus* | 2 | 2 |  |  |
| Uncharacterized protein OS=*Microbacterium sp.* AISO3 | 3 |  |  |  |
| Ribose-5-phosphate isomerase OS=*Micrococcus sp. HMSC067E09* | 3 | 2 |  |  |
| ATP-binding protein OS=*Micrococcus luteus* | 3 | 3 |  |  |
| Uncharacterized protein OS=*Micrococcus luteus* | 3 | 3 |  |  |
| 60 kDa chaperonin OS=*Rhodococcus qingshengii* | 4 |  |  | 2 |
| CsbD family protein OS=*Rhodococcus qingshengii* | 2 | 1 |  | 1 |
| Mycolyltransferase OS=*Rhodococcus qingshengii* | 2 | 1 | 1 | 1 |
| Aldehyde dehydrogenase OS=*Rhodococcus qingshengii* | 2 | 1 |  | 1 |
| Redoxin family protein OS=*Micrococcus luteus* SK58 | 1 |  | 2 | 1 |
| Chaperone protein DnaK OS=*Rhodococcus sp.* AD45 | 2 | 1 |  | 1 |
| NLPA lipoprotein OS=*Micrococcus luteus* | 3 | 1 |  |  |
| 2,3-bisphosphoglycerate-dependent phosphoglycerate mutase OS=*Micrococcus luteus* | 2 | 2 |  |  |
| Catalase OS=*Micrococcus luteus* |  |  |  |  |
| Electron transfer flavoprotein subunit alpha OS=*Rhodococcus qingshengii* | 2 |  |  | 1 |
| ABC transporter substrate-binding protein OS=*Rhodococcus erythropolis* | 2 |  |  |  |
| Ribose-phosphate pyrophosphokinase OS=*Micrococcus luteus* |  | 2 |  |  |
| Alanine racemase OS=*Micrococcus luteus* |  | 2 |  |  |
| Xaa-Pro aminopeptidase 1 OS=*Micrococcus luteus* |  | 2 |  |  |
| Nucleoside diphosphate kinase OS=*Micrococcus luteus* |  | 2 |  |  |

**Supplementary Table 2 All proteins identified within active IEX fractions by LC-MS analysis.** Highlighted proteins are present in all three IEX fractions analysed. The numbers in the columns represent the number of unique peptides that have been matched to the identified protein in that sample. The greater the number of matches the more certain the identification; 1 match is a possible identification, 2 – 3 matches a probable identification, 4 and over an almost certain identification. Origin species (OS).

| **Identified Proteins (32)** | **IEX1** | **IEX2** | **IEX3** |
| --- | --- | --- | --- |
|  |  |  |  |
| Uncharacterized protein (Fragment) OS=*Microbacterium sp.* AISO3 | 3 | 1 | 16 |
| Glyceraldehyde-3-phosphate dehydrogenase OS=*Micrococcus luteus* | 11 | 7 | 9 |
| Catalase OS=*Micrococcus luteus* | 7 | 4 | 14 |
| Catalase OS=*Micrococcus sp.* HMSC31B01 | 6 | 4 | 13 |
| PA domain protein OS=*Micrococcus luteus* | 6 | 6 | 2 |
| Uncharacterized protein OS=*Microbacterium sp.* AISO3 |  |  | 8 |
| Phosphate-binding protein PstS OS=*Micrococcus luteus* Mu201 | 3 | 3 | 3 |
| Dihydrolipoyl dehydrogenase OS=*Micrococcus luteus* | 4 | 2 | 4 |
| Putative phenylalanine aminotransferase OS=*Micrococcus luteus* | 3 | 2 | 3 |
| ATP-binding protein OS=*Micrococcus luteus* |  | 2 | 5 |
| Xaa-Pro aminopeptidase 1 OS=*Micrococcus luteus* | 2 |  | 5 |
| Uncharacterized protein OS=*Micrococcus luteus* SK58 | 2 | 2 | 1 |
| Amidohydrolase family protein OS=*Micrococcus luteus* SK58 | 2 | 1 | 3 |
| Chlorite O(2)-lyase OS=*Micrococcus luteus* | 2 |  | 3 |
| 2,3,4,5-tetrahydropyridine-2,6-dicarboxylate N-acetyltransferase OS=*Micrococcus luteus* | 2 | 1 |  |
| ABC transporter, substrate-binding protein, family 3 OS=*Micrococcus luteus* | 3 |  | 1 |
| Deoxyribose-phosphate aldolase OS=*Micrococcus luteus* | 2 | 2 |  |
| Hydrolase, alpha/beta domain protein OS=*Micrococcus luteus* | 1 | 2 |  |
| Acetyl-CoA C-acetyltransferase OS=*Micrococcus luteus* |  | 1 | 3 |
| Putative mycothione reductase OS=*Micrococcus luteus* |  |  | 4 |
| Glutamate dehydrogenase OS=*Micrococcus luteus* |  |  | 4 |
| Inosine-5'-monophosphate dehydrogenase OS=*Micrococcus luteus* |  | 2 | 1 |
| Thioesterase OS=*Micrococcus sp.* CH7 | 1 | 2 |  |
| Uncharacterized protein OS=*Micrococcus luteus* | 2 | 1 |  |
| Phosphoglycerate kinase OS=*Micrococcaceae bacterium* JKS001869 |  |  | 3 |
| Acetyl-CoA C-acetyltransferase OS=*Micrococcus luteus* |  |  | 3 |
| Thiol-disulfide isomerase OS=*Micrococcus luteus* |  | 2 |  |
| Probable cytosol aminopeptidase OS=*Micrococcus luteus* |  | 2 |  |
| Oxidoreductase, aldo/keto reductase family protein OS=*Micrococcus luteus* |  |  | 2 |
| Succinate-semialdehyde dehydrogenase OS=*Micrococcus luteus* |  |  | 2 |
| FHA domain protein OS=*Micrococcus luteus* |  | 2 |  |
| Uncharacterized protein (Fragment) OS=*Micrococcus luteus* | 2 |  |  |

Supplementary Table 3 A frame shift mutation and multiple non-synonymous coding mutations in the PA domain protein encoding gene are present in the inactive type strain *M. luteus* NCTC 2665 genome when compared to the active skin isolated *M. luteus* FAML strain.

The table summarises the resulting amino acid change and location. NSC: Non-synonymous coding, FS: Frame shift

| ***M. luteus* FAML allele** | ***M. luteus* NCTC 2665 allele** | **Mutation Type** | **Mutation Strength** | **Mutation Type2** | **Amino Acid Substitution** |
| --- | --- | --- | --- | --- | --- |
| A | C | NSC | MODERATE | MISSENSE | E114A |
| A | G | NSC | MODERATE | MISSENSE | T119A |
| C | T | NSC | MODERATE | MISSENSE | A124V |
| A | C | NSC | MODERATE | MISSENSE | E146A |
| C | G | NSC | MODERATE | MISSENSE | P175A |
| A | G | NSC | MODERATE | MISSENSE | K193E |
| G | C | NSC | MODERATE | MISSENSE | E249D |
| C | A | NSC | MODERATE | MISSENSE | H332N |
| A | G | NSC | MODERATE | MISSENSE | N346D |
| A | ACCCGGTGGCCATGGCCTTCAAGTCCGCCGCCGAGG | FS | HIGH |  | G396RWPWPSSPPPRP? |
| C | G | NSC | MODERATE | MISSENSE | D483E |
| A | C | NSC | MODERATE | MISSENSE | E564A |
| A | G | NSC | MODERATE | MISSENSE | M747V |
| G | A | NSC | MODERATE | MISSENSE | V775M |
| A | G | NSC | MODERATE | MISSENSE | T850A |
| T | C | NSC | MODERATE | MISSENSE | V858A |
| A | C | NSC | MODERATE | MISSENSE | K874Q |
| G | C | NSC | MODERATE | MISSENSE | E907D |
| A | G | NSC | MODERATE | MISSENSE | N916D |
| A | G | NSC | MODERATE | MISSENSE | T918A |
| C | T | NSC | MODERATE | MISSENSE | T918I |

| ***M. luteus* FAML** | ***M. luteus* NCTC 2665 Type strain** |
| --- | --- |
| CDS             48693..51695                       /locus_tag="248125_5_01657"                       /inference="ab initio prediction:Prodigal:2.6"                       /codon_start=1                       /transl_table=11                       /product="hypothetical protein"    /translation="MRKAEAMPQNPTPARRRRALAAAVAGASLVAAPALAVSATAVEL  PDGSTVSSPQGEVQVQQQFEDGRYFVVLKDQPSVTAPEAGAVPGAAPKAKFDPSHPRV  KNYEAKLQRQQEKVAKTHGAKAEISFQRAVNAFVAELTAEEAQEIAKDPAVLGVAPDE  QVAPDYSSTEFLGLPGKKGTWKSVYGKAENAGKGVVVGVIDSGIHPDNPFIDGQPVQP  LKGKAKVGVPYRTADGQIAVLKADGTTATAECETGPDFPASSCDSKLIGAYAFSEDFE  RFVPVDERAPEERISPLGVFSHGTHVATTILGNTGVEQTIDGDSFGEGAGVAPAAHLI  SYKICWEDTDPNTGGCYTSASVAAVEQAIENNVDVLNYSISGSNTSIVDPVAMAFKSA  AEAGIFVAASGGNSGPGPNTVNHGSPWLTTVAAETFSNELTATVQFSDGTQLRGASSA  RTGVGPAEVIHASEVAAGDAEAARLCLPGGLTDEAAGKIVLCERGVNARTEKSQVVEE  AGGVGMILVNTPSGSLDADIHAVPTVHMNDNGVIEKVKSSDLTATIVPGDTTGLPEDP  LPQIAGFSSRGPANAVNQELLKPDLAAPGVNVIAGVSPLDPDYHGNTFGLMSGTSMAS  PNLAGMATLLIGKYPAWSPMAVKSALMTTAGDVYNADGTVNTDNFATGAGSADPAAAA  RPGLVYESGKEQWDALLRGDIAGRDVNVPSLAIPDVVGSATVTRTVTALENGRWQFSA  NVPGFEMTASPAVLDLKAGQSADVELTVTRTDAAVNTWTHGSMSWTTAKGKAVPEVTS  PVTVKAKSATVTSAVEGSGATGSADVEITPGVTGELTPQVLGLGKVDSTVATATASNS  LVSSALAVSTVTVEEGTKSLVASINAGAAGADWDLYVITPEGKQLSRATAEESETLTI  ANPTPGAYTVVGHLYAANGGKDTGTLETLKLREDAGNLTVSPNPVPVTSGKATEATLS  WSGLTSGTWKGLVTWDAGITTDVTVQVP" | CDS             complement(54893..56701)                       /locus_tag="248135_Mluteusreference_01523"                       /inference="ab initio prediction:Prodigal:2.6"                       /codon_start=1                       /transl_table=11                       /product="hypothetical protein"    /translation="MAASGGNSGPGPNTVNHGSPWLTTVAAETFSNELTATVQFSDGT  QLRGASSARTGVGPAEVIHASEVAAGDAEAARLCLPGGLTEEAAGKIVLCERGVNART  EKSQVVEEAGGVGMILVNTPSGSLDADIHAVPTVHMNDNGVIEKVKSSDLTATIVPGD  TTGLPADPLPQIAGFSSRGPANAVNQELLKPDLAAPGVNVIAGVSPLDPDYHGNTFGL  MSGTSMASPNLAGMATLLIGKYPAWSPMAVKSALMTTAGDVYNADGTVNTDNFATGAG  SADPAAAARPGLVYESGKEQWDALLRGDIAGRDVNVPSLAIPDVVGSATVTRTVTALE  NGRWQFSANVPGFEVTASPAVLDLKAGQSADVELTVTRTDAAMNTWTHGSMSWTTAKG  KAVPEVTSPVTVKAKSATVTSAVEGSGATGSADVEITPGVTGELTPQVLGLGKVDSTV  AAATASNSLASSALAVSTVTVEEGTQSLVASINAGAAGADWDLYVITPEGKQLSRATA  DESETLTIADPVPGAYTVVGHLYAANGGKDTGTLETLKLREDAGNLTVSPNPVPVTSG  KATEATLSWSGLTSGTWKGLVTWDAGITTDVTVQVP"         CDS             complement(56656..57930)                       /locus_tag="248135_Mluteusreference_01524"                       /inference="ab initio prediction:Prodigal:2.6"                       /codon_start=1                       /transl_table=11                       /product="hypothetical protein"    /translation="MRKAEAMPQNPTPARRRRALAAAVAGASLVAAPALAVSATAVEL  PDGSTVSSPQGEVQVQQQFEDGRYFVVLKDQPSVTAPEAGAVPGAAPKAKFDPSHPRV  KNYEAKLQRQQAKVAKAHGAKVEISFQRAVNAFVAELTAEEAQAIAKDPAVLGVAPDE  QVAPDYSSTEFLGLAGKKGTWKSVYGKAENAGEGVVVGVIDSGIHPDNPFIDGQPVQP  LKGKAKVGVPYRTADGQIAVLKADGTTATADCETGPDFPASSCDSKLIGAYAFSEDFE  RFVPVDERAPEERISPLGVFSHGTHVATTILGNTGVEQTIDGDSFGEGAGVAPAANLI  SYKICWEDTDPDTGGCYTSASVAAVEQAIENNVDVLNYSISGSNTSIVDPVAMAFKSA  AEARWPWPSSPPPRPASSWPPPAATPAPARTP" |

Supplementary Figure 3 A representation of the PA domain protein sequence in the skin isolated *M. luteus* FAML and the corresponding sequences in the type strain *M. luteus* NCTC 2665. The gene encoding PA domain protein in the *M. luteus* NCTC 2665 genome contains a frame shift mutation which is predicted to result in two truncated proteins.
